# Thrombospondin-1/CD47 Interaction Regulates Th17 and Treg Differentiation in Psoriasis

**DOI:** 10.3389/fimmu.2019.01268

**Published:** 2019-06-04

**Authors:** Pedro Rodríguez-Jiménez, Pablo Chicharro, Mar Llamas-Velasco, Danay Cibrian, Laura Trigo-Torres, Alicia Vara, María Jiménez-Fernández, Javier Sevilla-Montero, Maria J. Calzada, Francisco Sánchez-Madrid, Hortensia de la Fuente, Esteban Daudén

**Affiliations:** ^1^Department of Dermatology, Instituto de Investigación Sanitaria Princesa, Hospital Universitario la Princesa, Universidad Autónoma de Madrid, Madrid, Spain; ^2^Department of Immunology, Instituto de Investigación Sanitaria Princesa, Hospital Universitario la Princesa, Universidad Autónoma de Madrid, Madrid, Spain; ^3^CIBER de Enfermedades Cardiovasculares, Institututo de Salud Carlos III, Madrid, Spain

**Keywords:** psoriasis, CD47, TSP-1, Th17, Treg cells

## Abstract

Accumulating evidence on the role of Thrombospondin-1 (TSP-1) in the immune response has emerged during the last years. In spite of the importance of TSP-1 not only as anti-angiogenic factor but also as an immunomodulatory molecule, studies on the role of TSP-1 in psoriasis have been neglected. TSP-1 and CD47 expression were analyzed in skin samples from psoriasis patients and control subjects using RT-PCR and immunofluorescence. Expression of these molecules was also evaluated in peripheral blood CD4+ T cells, moDCs, and circulating primary DCs. The functional role of TSP-1/CD47 signaling axis in psoriasis was assessed in Th17 and Treg differentiation assays. Additionally, small interfering RNA assays specific to TSP-1 were performed in CD4+ T cells and monocyte derived DC to specifically evaluate the function of this protein. Lesional skin of psoriasis patients expressed lower TSP-1 and CD47 mRNA levels compared to non-lesional skin or skin from controls. Immunofluorescence staining revealed decreased expression of CD47 in CD45+ dermal cells from psoriasis samples compared to control subjects. Peripheral CD4+ T cells and circulating primary DCs from psoriasis also expressed lower levels of CD47 compared to controls. Although no significant differences were detected in TSP-1 expression in CD4+ T cells and moDCs between patients and controls, TSP-1 expression in psoriasis patients inversely correlated with disease activity evaluated by the Psoriasis Area and Index Activity. Furthermore, exogenous TSP-1 inhibited Th17 differentiation and stimulated the differentiation of CD4+ T cells toward Treg cells. Furthermore, RNA interference specific for TSP-1 confirmed the role of this molecule as a negative regulator of T cell activation. Because of the impact of TSP-1/CD47 signaling axis in Th17 and Treg differentiation, a dysregulated expression of these molecules in the immune cells from psoriasis patients may favor the exacerbated inflammatory response in this disease.

## Introduction

Psoriasis is considered a chronic inflammatory autoimmune disease characterized by exacerbated proliferation and disturbed keratinocyte maturation, inflammatory dermal infiltrates and changes of the superficial microvasculature that result in an angiogenic phenotype (1). This condition arises from interactions between keratinocytes, infiltrating Interleukin (IL)-17-, IL-22-, and interferon (IFN)-γ-producing cells, inflammatory macrophages and dendritic cells (DCs) (2). Clinical investigations and experimental studies indicate that IFN-γ-producing T helper type (Th)1 cells and CD4^+^ Th17 cells accompanied by increased expression of IL-17A and accumulation of CD8^+^ cytotoxic T cells in psoriatic lesions cooperate at the interface of innate and adaptive immunity by activating keratinocytes to produce IL-17C. This cytokine together with other keratinocyte-derived mediators sustains chronic inflammation in psoriatic plaques (3–5). Although inflammation mediated by CD4^+^ Th17 T cells is considered an important trigger in the development of psoriasis, the precise underlying immuno-inflammatory mechanisms are still to be fully defined.

The multicellular protein thrombospondin-1 (TSP-1) is highly considered for its role in vascular health and disease. TSP-1 modulates vascular response and at pathologic levels promotes vascular dysfunction (6, 7). TSP-1 regulates multiple cellular events involved in tissue repair including cell adhesion, migration, proliferation, extracellular matrix expression and organization, and regulation of growth factor activity (8). This is possible due to its multi-domain structure which interacts with several cell receptors and matrix proteins (9). Despite TSP-1 is mostly known for its role in modifying the tumor micro-environment through its anti-angiogenic properties (10), growing evidence about the role of TSP-1 in immune response has emerged in the last decade (11–13).

TSP-1 is known to regulate the conversion of tumor growth factor β (TGF- β) from latent to an active form (14). On the other hand, TGF- β1 is a potent keratinocyte growth inhibitor, and its signaling pathway is downregulated in psoriatic skin leading to abnormal cell proliferation due to a functional decrease in growth regulation (15, 16). Independently of TGF- β regulation, TSP-1 may also have suppressive properties by means of its interaction with CD47 in T cells (17). Particularly, in T CD4+ lymphocytes TSP-1/CD47 interaction promotes an anti-proliferative effect as well as the generation of human peripheral regulatory T (Treg) cells (18–20). Furthermore, when released after the inflammatory response, the interaction between TSP-1 and CD47 reduces inflammation linked to T cell activation (20, 21). Interestingly, methotrexate stimulates the expression of endogenous TSP-1 in primary human T cells, which could explain its beneficial effects in psoriasis, due to its immunoregulatory effects (22). Human immature monocyte-derived DCs (moDCs) as well are known to spontaneously produce TSP-1, this being enhanced by microbial stimuli (11). It has also been shown that increased TSP-1 levels during DC activation and its interaction with CD47 and CD36, negatively regulate IL-12, tumor necrosis factor α (TNF- α) and IL-10 release (23–25). Although our knowledge about the functional relevance of TSP-1 in psoriasis is scarce, a defective expression of TSP-1 in keratinocytes from psoriasis patients correlates with a higher angiogenic response (26). In this paper, we sought to the role of TSP-1/CD47 signaling axis in the development and maintenance of psoriatic lesions. Our results showed that TSP-1 binding to its receptor CD47 was able to inhibit the differentiation of Th17 cells and favor differentiation of CD4+ T cells toward Treg cells. Therefore, a diminished expression of TSP-1 and its receptor CD47 in immune cells from psoriasis patients may promote the exacerbated inflammatory response characteristic of this disease.

## Methods

### Patients

This study was approved by the Institutional Review Board (IRB)/Independent Ethics Committee of Hospital de la Princesa according to the Declaration of Helsinki Principles. After giving informed consent, 20 healthy individuals and 30 untreated psoriatic patients were enrolled. Patients were eligible for the study if they had a Psoriasis Assessment Severity Index (PASI) ≥ 8. The following washout periods were established: 14 days for topical corticosteroids, 28 days for systemic treatment including corticosteroids, methotrexate, cyclosporine, acitretin, or phototherapy and 3 months for biologic agents. From each psoriasis patient, two non-sun-exposed cutaneous biopsies (10 mm) were taken, one from lesional psoriatic skin and other from apparently healthy skin (non-lesional skin). At the same time, 20 ml of peripheral venous blood were obtained. Normal leftover skin samples and peripheral venous blood samples were obtained from 20 surgical patients. Each biopsy was cut in half; one piece was snap frozen for RNA isolation and the other one was embedded in OCT™ compound (Sakura Finetek, Zoeterwoude, NL) and stored at −80°C until processing for immunofluorescence.

### Expression of TSP-1 and CD47 mRNA Levels by RT-PCR

TSP-1 and CD47 mRNA expression levels were determined by reverse transcription polymerase chain reaction (RT-PCR). Total RNA was isolated from skin samples, peripheral blood CD4+ T cells and moDCs using TRIzol reagent (Invitrogen) following the manufacturer's instructions. One microgram of RNA was reverse-transcribed to cDNA and amplified with the specific primers pairs (TSP-1 forward GCC ACA GTT CCT GAT GGA G, reverse CCA TGG AGA CCA GCC ATC; CD47 forward TCC ACA GCA CAG CCA AGG T, reverse TCG CAG ATG ACT TGA GAG TGA AC) using GoTaq qPCR Master Mix (Promega, WI USA). The data were analyzed using StepOne Plus Software (Applied Biosystems, Carlsbad, CA). TSP-1 and CD47 mRNA levels were normalized to GAPDH levels.

### Immunofluorescence Staining

Skin OCT sections of 5 μm were fixed (formaldehyde 4%), permeabilized (Triton X-100 0,2%) and blocked with 100 μg/ml human gammaglobulin (Sigma-Aldrich, St. Louis MO, USA) and a 1:100 dilution of donkey serum (Sigma-Aldrich) in phosphate buffer solution (PBS). Skin sections were then incubated over-night with 5 μg/ml sheep anti-human CD47 (R&D systems, Cat. AF4670) and mouse anti-human CD45, followed by donkey anti-sheep (DAS) Alexa Fluor 488 and donkey anti-mouse (DAM) Alexa Fluor 555. Finally, cell nuclei were counterstained with DAPI. Negative controls were performed with omission of the primary antibody. Sections were examined with a Leica DMR immunofluorescence microscopy under the same acquisition conditions. Images were analyzed using the ImageJ sowftware (http://imagej.softonic.com). For the analysis of CD47 expression, fluorescence intensity was determined in regions of interest (ROIs) drawn on CD45+ cells.

### Peripheral Blood CD4+ T Cells and Monocyte Derived DCs (moDCs)

Peripheral blood mononuclear cells (PBMNCs) were obtained by density gradient centrifugation and then CD4+ T cells were isolated by negative selection using magnetic microbeads (Miltenyi Biotec, Bergisch Gladbach, Germany). For moDCs, PBMNCs were allowed to adhere for 30 min at 37°C, and plastic adhered cells were cultured for 5 days in complete media supplemented with 500 U/ml GM-CSF (Peprotech) and 10 ng/ml IL-4 (R&D systems). On day 6, 10 ng/ml LPS were added and after 24 h cells were harvested.

### Th17 and Treg Differentiation Assays

For Th17 differentiation, isolated CD4+ T cells were cultured for 10 days with anti-CD3 (BioLegend Cat# 300314, RRID:AB314050) plus anti-CD28 mAbs (BD, Cat#555725) (at 5 μg/ml and 2 μg/ml, respectively) in the presence of the combination of cytokines and blocking antibodies appropriate for polarization: rhIL-6 h IL-1β (10 ng/ml), rhIL-23 (20 ng/ml), rhTGF-β1 (2 ng/ml), anti-IFN-γ (10 μg/ml) and anti-IL-4 (10 μg/ml). For Treg polarization, cells were cultured with TGF-β (5 ng/ml) and IL-2 (20 U/ml) (all cytokines from R&D systems) for 5 days. Where indicated hTSP-1 (5 μg/ml), mouse anti-human CD47 (1 μg/ml) clone B6H12.2 **(**Abcam Cat# ab3283, RRID:AB303671) or IgG1 isotype control were also added. Percentage of IL-17+ and FoxP3+ CD25+ cells (Treg) was measured in a FACS Canto cytometer and analyzed with FlowJo software.

### siRNA Transfection

Transfections were carried out using 4-D Nucleofector and P3 primary cell kit (Amaxa Lonza, Köln, Germany). Unstimulated CD4+ T cells or immature moDCs (1 × 10^6^ cells) were transfected with TSP-1 specific siRNA (50 pmol) selecting the EO-115 protocol from the 4-D Nucleofector for CD4+ T cells and the protocol CM-120 for moDCs. Immediately after nucleofection, cells were incubated in X-VIVO 15 (Lonza, Belgium) medium.

### Mixed Leukocytes Reaction (MLR) Assay

CD4+ T cells were co-cultured with moDCs from a different donor in 96-well U-bottom plates in X-VIVO 15 medium. The ratio of DCs and CD4+ T cells was 1:5. CD4+ T cells were preloaded with CellTrace Violet (Invitrogen, by Thermo Fisher Scientific, OR USA) to follow cell proliferation. Cells were cultured for 5 days, then intracellular cytokine production and CellTrace Violet dilution was assessed by flow cytometry.

### Treg Suppression Assays

After 5 days of differentiation, Treg cells were isolated using magnetic beads and added to MLR cultures (ratio 1:2). Responder CD4+ T cells were from the same donor of Treg cells. Immediately before the co-culture, responder T cells were loaded with CellTrace Violet, while Treg cells were loaded with CFSE (Invitrogen). After 5 days of culture, proliferation of responder cells was evaluated by flow cytometry.

### Statistical Analysis

Data were analyzed with GraphPad Prism (GraphPad Software, San Diego, CA, USA). One Way Anova, Tukey test and Mann-Whitney *U*-test were used as appropriate. Where indicated, Wilcoxon signed rank test was used to paired data. Data from T cell differentiation assays were analyzed using Friedman test and Dunn's Multiple Comparison. The Spearman test was used for correlation analysis. Differences were considered significant at *p* < 0.05.

## Results

**Figure 1:**
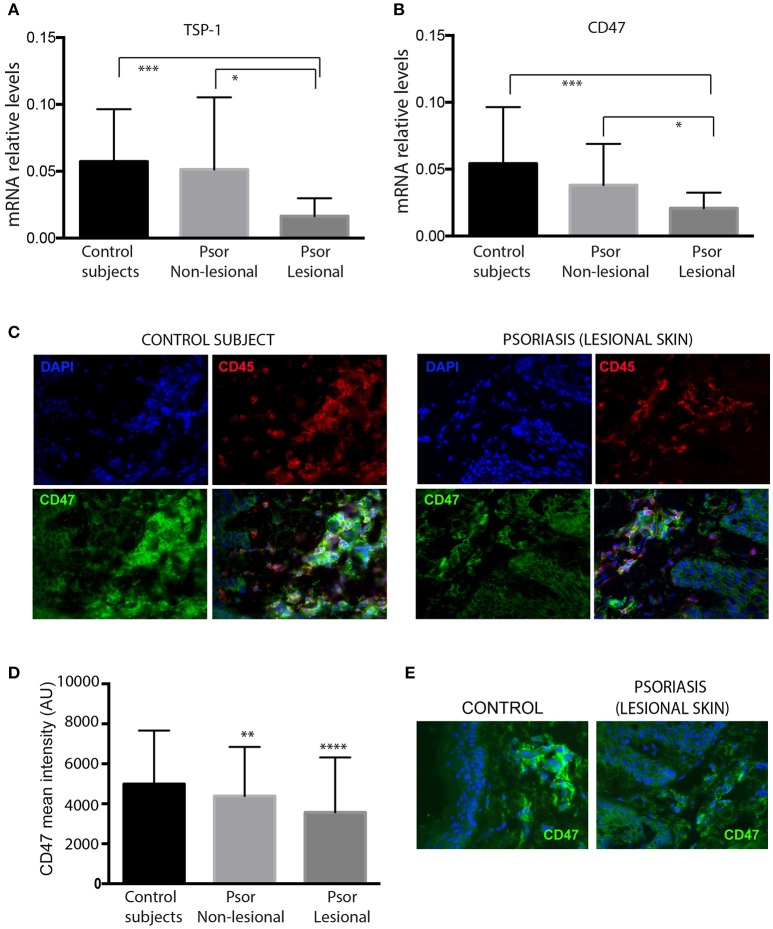
Skin samples from psoriasis patients express lower levels of TSP-1, and CD47 compared with healthy controls. mRNA levels of TSP-1 **(A)** and its receptor CD47 **(B)** were analyzed by RT-PCR in skin samples from 26 psoriasis patients and 20 healthy controls. GAPDH expression was used to normalize gene expression. Data were analyzed by one-way ANOVA followed by Tukey's multiple comparisons test, ^***^p < 0.001, ^*^p < 0.05. **(C)** Double immunofluorescence staining of CD47 (green) and CD45 (red) in a representative skin sample from control subjects (left panels) and lesional skin from psoriasis patients (right panels) is shown. Nuclei were counterstained with DAPI (blue). **(D)** For quantification of immunofluorescence staining, fluorescence intensity of CD47 in CD45+ dermal cells was calculated using Image J software. **(E)** Representative expression of CD47 (green) in skin samples from control subjects and psoriasis patients. Graphs represent mean ± SD. Differences between groups were determined by one-way ANOVA followed by Tukey's multiple comparisons test, ^****^p < 0.0001, ^**^p ≤ 0.001.

Expression of TSP-1 and CD47 was analyzed by RT-PCR in skin samples from psoriasis patients and healthy controls. Our data showed that lesional skin from psoriasis patients express lower levels of TSP-1 and CD47 compared to non-lesional skin or skin from control subjects (Figures 1A,B). Immunofluorescence assays showed that CD47 is expressed in the dermis and epidermis of both control and psoriasis skin samples. Furthermore, double immunostaining with CD45 identified the expression of CD47 in dermal leukocytes (Figure 1C). Quantitative analysis demonstrated diminished levels of CD47 in CD45+ dermal cells of psoriasis patients compared to cells from non-lesional skin or cells from control subjects (Figure 1D). Conversely, we did not observe any difference in the levels of CD47 in keratinocytes between psoriasis patients and healthy controls (Figure 1E).

**Figure 2:**
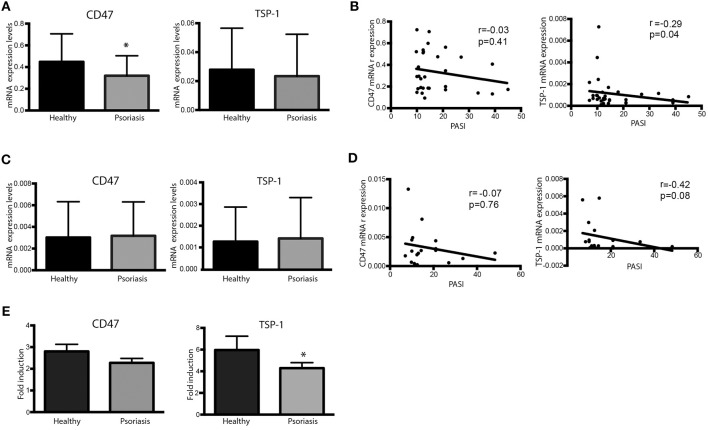
Dysregulated expression of CD47 and TSP-1 in peripheral blood CD4+ T lymphocytes and moDCs. **(A,B)** CD4+ T cells from psoriasis patients (*n* = 30) and healthy controls (*n* = 18) were isolated from peripheral blood using magnetic microbeads**. (A)** CD47 and TSP-1 expression was analyzed using real-time PCR. GAPDH expression was used to normalize data. Differences between groups were analyzed using Mann-Whitney *U*-test. **(B)** Correlation between the expression of CD47 and TSP-1 in CD4+ T cells and PASI determined by Spearman test. **(C)** Basal expression of CD47 and TSP-1 in non-stimulated moDCS from patients and controls. Monocytes were isolated from peripheral blood using immune-magnetic bead and differentiated to DCs during 5 days in the presence of IL-4 and GM-CSF. **(D)** Correlation between the expression of CD47 and TSP-1 in moDCs cells and PASI determined by Spearman test. **(E)** Fold induction of CD47 and TSP-1 expression levels in moDCs in response to LPS. moDCs were stimulated for 24 h in the presence of LPS (10 ng/ml), then RNA was isolated and the expression of TSP-1 and CD47 analyzed by RT-PCR, calculating their fold induction as LPS/basal expression (*n* = 18). Differences were tested by Mann-Whitney *U*-test, ^*^p < 0.05.

Expression of CD47 and TSP-1 was also analyzed in peripheral blood CD4+ T cells and monocyte-derived DCs (moDCs). Our data showed that peripheral CD4+ T cells from psoriasis patients expressed lower levels of CD47 compared to healthy controls (Figure 2A). Although our results did not prove significant differences in TSP-1 mRNA levels between healthy subjects and psoriasis patients (Figure 2A), statistical analysis showed a negative correlation between TSP-1 expression and Psoriasis Assessment Severity Index (PASI) (Figure 2B). However, no correlation between CD47 expression and PASI was observed (Figure 2B).

The expression of TSP-1 and CD47 was also analyzed in moDCs under basal conditions or following activation with LPS. No significant differences were observed in the basal expression of TSP-1 and CD47 between psoriasis patients and healthy controls (Figure 2C). However, similarly to our findings in peripheral CD4+ T cells, TSP-1 expression levels in immature moDCs negatively correlated with PASI (Figure 2D). Interestingly, TSP-1 induction in response to LPS was lower in moDCs from psoriasis patients compared to controls, while no significant differences in CD47 expression levels were found (Figure 2E).

**Figure 3:**
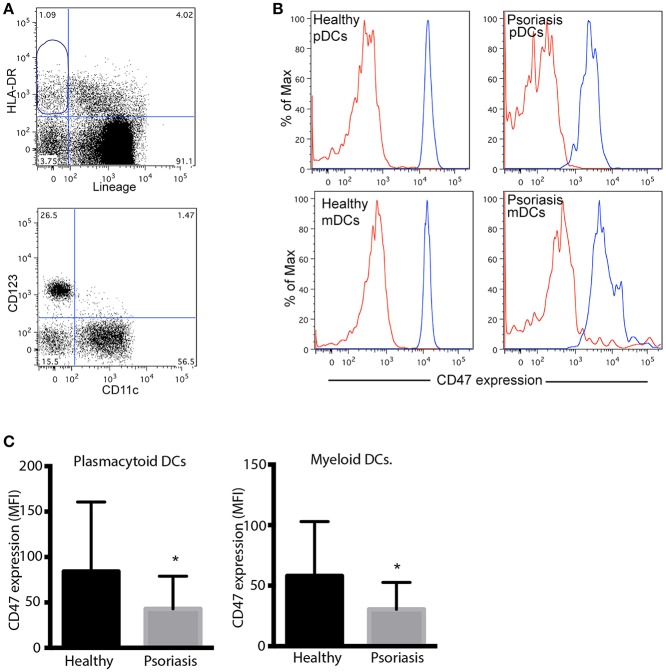
Peripheral blood plasmacytoid and myeloid DCs from psoriasis patients express low levels of CD47. **(A)** Gating strategy for the detection of pDCs and mDCs. Dendritic cells were identified as HLA-DR positive, Lineage (CD3, CD14, CD20, CD56) negative, CD47 expression was then determined in CD123+ and CD11c+ by flow cytometry. **(B)** Representative histograms of CD47 expression in pDCs and mDCs from one patient and one control subject. Red and blues lines indicates isotype control and CD47 expression, respectively. **(C)** CD47 expression was calculated as mean fluorescence intensity (MFI) of CD47/MFI isotype control. Bars represent mean ± SD from 18 psoriasis patients and 10 control subjects. Differences were analyzed by the Mann-Whitney *U*-test, ^*^p < 0.05.

CD47 protein levels were evaluated by flow cytometry in plasmacytoid DCs (pDCs) and myeloid DCs (mDCs) from psoriasis patients and healthy controls. DCs were identified as HLA-DR^+^ Lineage^−^ (CD3, CD14, CD20, CD56) cells and then selected according to CD123 and CD11c expression (pDCs and mDCs, respectively) (Figure 3A). Although CD47 protein levels were high in both pDCs and mDCs, we did not detect any significant difference in the expression of CD47 between both cell types (Figure 3B). However, both subsets of circulating DCs from psoriasis patients expressed significantly lower levels of CD47 compared to cells from healthy subjects (Figures 3B,C).

**Figure 4:**
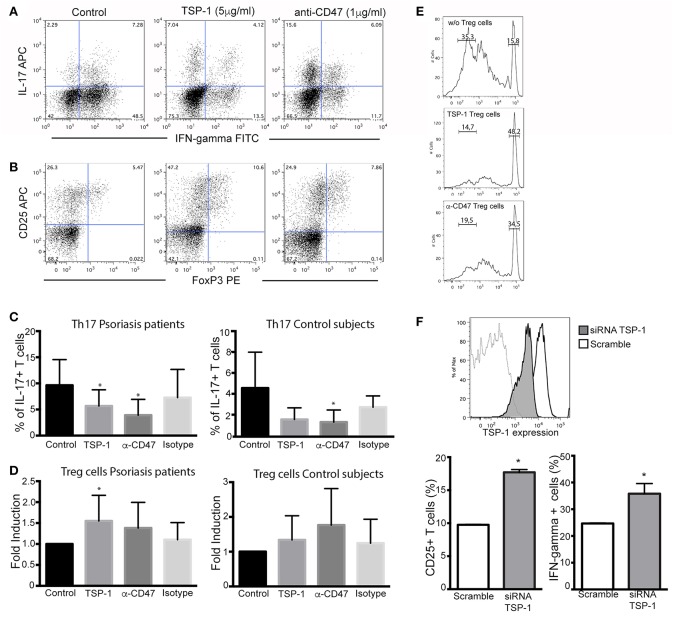
Functional effects of TSP-1 in CD4+ T lymphocytes from psoriasis patients. **(A,B)** Representative dot plots from Th17 **(A)** and Treg differentiation **(B)** assays in the presence of TSP-1 and anti-CD47 mAb. CD4+ T cells were isolated from peripheral venous blood from psoriasis patients using magnetic beads. Cells (1 × 10^6^/ml) were differentiated to Th17 in the presence or absence of human anti-CD47 mAb 1 μg/ml (left) or human TSP-1 5 μg/ml (center). **(C)** Percent of IL- 17+ T cells from psoriasis patients (*n* = 13) and control subjects (*n* = 5) evaluated by flow cytometry after 10 days of culture in the presence of TSP-1, anti-CD47 mAb, or IgG1 isotype control (1 μg/ml). Data were analyzed using Friedman test and Dunn's multiple comparison test, ^*^p < 0.05. **(D)** Differentiation of Treg cells in the presence of anti-CD47 mAb (1 μg/ml), TSP-1 (5 μg/ml) or IgG1 isotype control (1 μg/ml) from psoriasis patients (*n* = 9) and control subjects (*n* = 6). Graphs show the fold induction of FoxP3+ CD25+ cells in the presence of the indicated stimuli compared to the cells cultured only with the differentiation cocktail. **(E)** Treg suppression assay, Treg cells differentiated in the presence of TSP-1 or anti-CD47 mAb were isolated by immunomagnetic beads and added to mixed leukocytes reaction (MLR) cultures. Upper histogram corresponds to control culture (moDCs plus responder T cells), central and lower histograms correspond to MLR cultures plus Treg cells that were differentiated in the presence of TSP-1 or anti-CD47 mAb (histograms are representative of two independent experiments). **(F)** Knockdown of TSP-1 favors T cell activation via TCR. Histogram showing the knockdown efficiency of TSP-1 siRNA. Briefly, unstimulated CD4+ T cells were transfected by nucleofection with TSP-1 specific or control siRNA (50 pmol), 24 h later T cells were stimulated with anti-CD3/CD28 + IL-2 during 48 h. CD25 expression and IFN-γ production were evaluated by flow cytometry data. Data correspond to one out of two independent experiments performed by triplicate. Differences were tested by Mann-Whitney test, ^*^p < 0.05.

To evaluate the functional role of CD47 and TSP-1 in psoriasis inflammatory response we performed Th17 and Treg differentiation assays on peripheral CD4+ T cells from psoriasis patients and healthy controls. CD4+ T cells were incubated with the corresponding cocktail of cytokines, and in the presence of human TSP-1 or anti-CD47 mAb that mimics TSP-1 binding (20) (Figures 4A,B). Our results demonstrated a clear and significant reduction in the percentage of IL-17+ cells when these cells were cultured in the presence of TSP-1 or anti-CD47 antibody (Figures 4A,C) in psoriasis patients (Friedman test *p* = 0.003). Similar to our data from psoriasis patients, TSP-1 and anti-CD47 reduced the percentage of IL-17+ cells in cell cultures from healthy donors (Friedman test *p* = 0.007) (Figure 4C). In addition, we assessed whether TSP-1 or anti-CD47 mAb were able to affect Treg differentiation. Our results showed that the addition of TSP-1 induced a significant increment in the percentage of Treg (CD25+ FoxP3+) cells in samples from psoriasis patients (Friedman test *p* = 0.03) (Figures 4B,D). Although we observed a tendency for anti-CD47 to favor Treg differentiation, differences were not significant (Figure 4D). In order to analyze the functionality of CD25+ FoxP3+ cells, we tested their ability to suppress T cell proliferation. Our data showed that CD25+ FoxP3+ cells differentiated in the presence of TSP-1 or anti-CD47 mAb were able to inhibit the proliferation of T cells in a mixed leukocytes reaction (MLR) (Figure 4E). To confirm the role of TSP-1 in CD4+ T cell activation, siRNA-mediated silencing was performed. The levels of exogenously expressed TSP-1 were monitored by flow cytometry. As shown in Figure 4F, protein levels dropped in the presence of siRNA targeted to TSP-1. Both CD25 expression and IFN-γ production were clearly augmented in T cells transfected with TSP-1-specific siRNA, when they were activated via TCR (Figure 4F).

**Figure 5:**
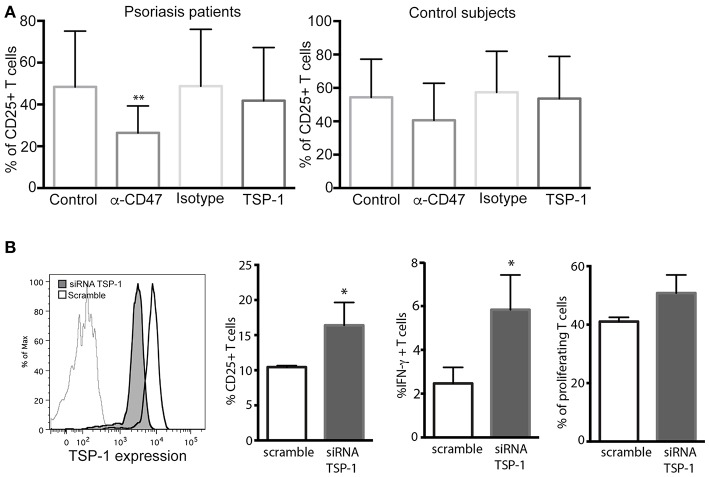
CD47/TSP-1 signaling prevents CD4+ T cell activation in an antigen presentation assay. **(A)** CD25 expression from CD4+ T cells after antigen presentation, monocyte-derived DCs from psoriasis patients (*n* = 8) and healthy controls (*n* = 3) were cultured for 3 days with autologous peripheral blood CD4+ T cells in the presence of staphylococcal enterotoxin E. Where indicated, anti-CD47 (1 μg/ml), IgG1 isotype control (1 μg/ml), or TSP-1 (5 μg/ml) were added. Then, expression of CD25 was evaluated using flow cytometry. Data correspond to mean ± SD and differences were tested using Friedman test, ^**^p < 0.01. **(B)** Knockdown of TSP-1 of moDCs augments the activation of T cells following Ag presentation. Histogram showing the knockdown efficiency of TSP-1 siRNA. Briefly, immature moDCs were transfected by nucleofection with TSP-1 specific or control siRNA (50 pmol), after 48 h moDCs were stimulated with LPS (10 ng/ml) during 12 h and then co-cultured with heterologous CD4+ T cells. Data correspond to one out of three experiments performed by triplicate. Differences were analyzed using Mann-Whitney test, ^*^p < 0.05.

Finally, the role of TSP-1/CD47 signaling axis was evaluated in antigen (Ag) presentation assays with autologous co-cultures of moDCs with CD4+ T lymphocytes and using the expression of CD25 as a marker of activation. These assays showed that the anti-CD47 mAb clearly prevented the activation of CD4+ T cells during Ag presentation (Figure 5A) in cells from psoriasis patients. However, the addition of human TSP-1 had no significant effects on CD25 expression (Figure 5A). No significant differences were observed in the co-cultures of cells from control subjects (Figure 5A). These findings contrast with those observed in T cell differentiation and activation (Figure 4). The effect of exogenous TSP-1 could be dose dependent, and it is also important to remark that anti-CD47 (clone B6H12.2) not only mimics TSP-1 binding but blocks as well the binding of SIRP alpha (27) expressed in DCs. Thus, to elucidate whether or not TSP-1 expressed by DCs was playing a role in CD4+ T cell activation, endogenous expression of TSP-1 was silenced in moDCs by siRNA assays. T cell activation in MLR assays, evaluated as CD25 expression, IFN-γ production and proliferation, was augmented in the presence of moDCs transfected with TSP-1 siRNA (Figure 5B). These data demonstrate that TSP-1 expressed by moDCs is involved in the activation of CD4+ T cells.

## Discussion

TSP-1/CD47 signaling axis has been considered an important sensor to maintain homeostasis and regulate innate and adaptive immune responses (28). In this study we identified a defective expression of CD47 not only locally in dermal leukocytes, but also in CD4+ T lymphocytes, pDCs, and mDCs from peripheral blood. The impact of CD47 deficiency in skin inflammation has been studied in animal models of contact hypersensitivity (CHS). An exacerbated T cell-mediated CHS response and prolonged inflammation indicate the importance of CD47 in different phases of the inflammatory response (21, 29). Moreover, a role during disease resolution in CHS has been observed in TSP-1 deficient mice (21). TSP-1 is present in low amounts in almost every tissue, but it is rapidly and transiently increased under stress or in damaged tissues in response to inflammatory signals (30). In healthy human skin, TSP-1 is produced by basal epidermal keratinocytes and is deposited in the dermo-epidermal basement membrane zone, contributing to the barrier that prevents the growth of blood vessels into the dermis. On the contrary, TSP-1 expression is downregulated in diseased skin, such as in skin squamous cell carcinomas (31) and in keratinocytes from psoriasis patients (26). T cells, macrophages and DCs may produce and express surface-bound TSP-1 (32–34). Our present results confirmed that TSP-1 levels are decreased in the skin of psoriasis patients, although no differences were detected in the expression of TSP-1 in peripheral CD4+ T cells or moDCs between patients and controls, However, we have shown that moDCs from psoriasis patients failed to upregulate TSP-1 expression in response to LPS. This is in agreement with the increase of TSP-1 in TLR-activated DCs vs. immature DCs observed by other authors (11). Thus, TSP-1 might be considered an important brake in psoriasis and this could be mediated by its dual effect impairing angiogenesis in the skin and regulating Th17 and Treg cell differentiation. Results from *in vitro* experiments have previously demonstrated that exogenously added TSP-1 or CD47-binding TSP-1 peptide inhibit IL-12 secretion by monocytes in response to T cell-dependent stimuli (35). Moreover, TSP-1 co-stimulates TCR-activated T cells through its interaction with α4β1 integrin (17). Our data demonstrate that TSP-1-CD47 interaction or the incubation with anti-CD47 mAb inhibits the differentiation of Th17 cells and favors CD4+ T cells differentiation into Treg cells from psoriasis patients. Topical administration of a CD47-binding TSP-1 peptide during the development of an ocular inflammation has been described to attenuate clinical symptoms of Sjögren syndrome-associated dry eye and augment FoxP3 expression (36). This is in agreement with previous results suggesting that TSP-1-mediated activation of TGF-β, as well as its binding to CD47 may create an anti-inflammatory environment in certain immune privileged sites such as the eye (37). It is well-established that CD47/SIRP alpha interaction negatively regulates antigen presentation (27). The anti-CD47 (clone B6H12.2) used in our functional assays, has been described to mimic TSP-1 binding but to also block the binding of CD47 with SIRP alpha. We cannot rule out that the blockade of CD47/SIRP alpha interaction is involved in the effects observed with anti-CD47 mAb. However, in line to previously published data this blockade would lead to a higher T cell activation instead of the inhibition detected in our assays (25, 27, 38). In the case of effects of exogenous TSP-1, it is difficult to reproduce the physiological concentration in that particular context. However, TSP-1 silencing assays clearly show that TSP-1 expressed by DCs acts as a negative regulator of immune response.

Together these data suggest that TSP-1 and its receptor CD47 may have a role in the exacerbated inflammatory response characteristic of psoriasis. The link between these molecules and the angiogenesis and immune regulation raises the possibility that they could be evaluated as activity markers in psoriasis.

## Ethics Statement

Comité de Ética de La investigación con Medicamentos del Hospital Universitario de la Princesa. All patients signed and Informed Consent

## Author Contributions

PR-J: acquisition and analysis data and drafting the manuscript. PC: acquisition data and drafting the manuscript. ML-V: analysis and drafting the manuscript. DC: design experiments. LT-T and AV: acquisition data. MJ-F and JS-M: acquisition data, prepared figures and interpreted result. MJC: drafting the article and interpretation of data. FS-M: drafting the article and data interpretation. HdF and ED: design of the work, interpretation, critical revision of the manuscript, and final approval.

### Conflict of Interest Statement

The authors declare that the research was conducted in the absence of any commercial or financial relationships that could be construed as a potential conflict of interest.
